# Annotations

**Published:** 1889-02-09

**Authors:** 


### ANNOTATIONS.

**Extract of "Horse."**

It is stated that a curious trade has sprung up in East Kent. Old and worn out horses are collected and shipped off to Holland, there to be killed and converted into "extract of beef." An article of this kind now in the market, which has attained considerable popularity, is said to consist entirely of "extract of horse." Mules are also in great request. One old mule, which was sold by its owner for £2 10s. in Ashford Market three weeks ago, was resold to an exporting agent for £15, and on reaching Flushing fetched the handsome figure of £22. There is absolutely nothing at all alarming in these statements, even if they be all quite true. Horse and mule are excellent food for men and women. They do not possess quite the same flavour as cow and sheep; but whether the flavour is worse or better must be entirely a matter of taste. It is evident that the soup or extract which is produced from them can be made pleasant to the palate, otherwise it could have attained no widespread popularity. Of course, whilst there is no objection to horse as horse, or even to mule as mule, there may be very strong objections to old horses and old mules, which in addition to being worn out may also be diseased. The objection is somewhat diminished by the fact that the animals are not sold in bulk for eating as joints, but are reduced by a thorough process of cooking to a liquid extract. This extract, as we have said, is evidently palatable, and it is probably harmless. Perhaps also the manufacturers are shrewd enough to understand that if they wish to retain their trade they must continue to produce a good article. Impartial science sees no more objection to "extract of horse" than to "extract of cow." But though science makes no objection, sentiment makes a very strong protest. "It is horrid," as ladies say, to think of the old coach horse, or even the family donkey, kidnapped, floated off to the Continent, killed, skinned, quartered, and boiled down, to be finally returned to the table of his mistress or to the nursery of his old friends the children, all done up in tins, and labelled "Concentrated essence of the best American beef." "Imagination will not forward with the picture," as Carlyle has it. What is to be done? The remedy is very simple. Let every lady who does not wish to dine off "mule soup" or "extract of coach-horse," have all her beef-tea made at home. There is a tendency in the present day to make nothing at home which can be bought in a shop. The tendency is idle and bad. Ladies should put a stop to it. Let everything be made at home which can be made. We shall then all know for ourselves what we are eating and drinking.

**"After Dinner Walk a Mile."**

"After dinner sit awhile, after supper walk a mile." That was suitable advice for the "good old times" when dinner was taken at noon. "The wise man changes his mind." We moderns have changed ours, and our habits too. The couplet may be changed to suit the new circumstances. "After luncheon sit awhile, after dinner walk a mile." This advice is by no means universally followed. It may be doubted whether it is universally given or believed in. One thing, however, is certain: the "mile," and much more, ought to be walked, sometime during the twenty-four hours. Nay, it must be walked if health is to be maintained. Indoor air cannot be breathed all day long without serious injury, nor can a sufficient measure of physical exercise be dispensed with. Nature is stronger than all the doctors and drugs in the world, and she will not let a man be well who persistently disobeys her. She has made our limbs for movement and our lungs for pure air. If we do not use the limbs sufficiently and breathe enough of perfectly pure air, she insists upon storing up quantities of poisonous waste in the system, and makes the arms and legs as limp as a jelly-fish. Men of business and professional men seem to have no time for walking and "taking the air" except in the evening. But how can a man walk after a heavy dinner? Most true; and therefore a man should not eat a "heavy dinner" habitually. Whether he walk or not, the "heavy dinner" will do him nothing but harm, and all the more harm if he does not walk. Most men eat a good meat lunch. Many take both meat and pudding: in fact, to all intents and purposes they "dine." They do not then need a heavy meal in the evening. After a substantial luncheon at one, a moderate dinner at six or seven is all that is required. If such a meal be taken, followed about eight o'clock by a cup of hot coffee, the man who has not been overworked during the day should feel perfectly fresh for a walk at half-past eight. If he then goes out and walks until half-past nine, he will soon begin to find his walk a great pleasure, and the advantage to his health will be very marked indeed. Does he fear the "night air"? That is nonsense. Night air is as good as any other air, except that it is a little colder. He can provide against that by wrapping up a little more. For getting rid of the cares of the day, for producing a pleasant sense of relaxation, for purifying the blood, for raising the spirits, for encouraging sound and refreshing sleep, there is nothing better than an evening walk after a moderate dinner. To those who have not practised the habit, the first few walks may prove fatiguing and disappointing; but let them give it a fair trial. Perseverance will amply justify what some may consider rather novel advice.

**"Dried-up" Pensioners.**

At the present moment a certain class of women—to wit, sick nurses—are much exercised in mind on the subject of their relative longevity. To live long or not to live long? that is the question. One or two clever and experienced nurses—whose experience, however, has been both limited and unfortunate—have come to the conclusion that nurses do not live long, and never can be expected to live long. They wear themselves out by the anxiety and worry of their duties, their want of sufficient holidays, and the mental strain of their unprosperous worldly condition. These writers seem to have arrived at the general conclusion that sick nurses are like the horses of the tramway companies—used up at full pressure by the public so long as their health and strength will stand it, and then, also like the "tram" horses, cast aside to earn a precarious living as they can, or to starve and die. That is a dismal picture. But is it a true representation of the facts? Let us confess frankly and freely that up to the present time it has not been possible to collect such a number and variety of facts as will warrant a broad, scientific, and permanent conclusion. But some facts there are which will at least indicate what is probable in the matter, and which it is safer to follow than to be satisfied with mere guesswork. In brief, a certain society has for some years been engaged in the meritorious work of pensioning off trained nurses. It is a small society, which may probably some day affiliate itself to the larger institution, the National Pension Fund. That society finds that trained nurses who, before old age, have the prospect of a pension, and in old age realise that prospect, live as a rule to very advanced ages, and greatly enjoy the repose, quiet, and security of their declining years. In practising the art of taking care of others, they have unconsciously acquired that of taking care of themselves. They are able to avoid or to treat for themselves many of the ailments which distress old age and shorten life—such, for example, as bronchitis, asthma, heart weakness, and the like. Hence it has been found that after retirement from the active duties of their profession at fifty-five or sixty, some who seemed to be in the last stage of debility and premature decay have gradually, under the influence of rest and modest comforts, seemed to develop a new constitution, and to "take a new lease of life." Not a few who appeared quite incapable of living beyond the age of sixty or sixty-five have long passed the maximum fourscore of the Psalmist, and their old age, thanks to their pension, has been neither "labour" nor "sorrow." These persons seldom die of those acute diseases, such as bronchitis and pneumonia, which are the peculiar enemies of old age. On the contrary, they lose their strength and vigour by such slow degrees that they are hardly conscious of it. Their friends see them getting a little thinner, a little older-looking, a little more "dried up" year by year, but still living on in quiet pleasure, awaiting without dread the slow but not unkindly approach of the last enemy. This process of "drying up" in old age is especially common among women, and in none is it so frequently seen as in those who have an assured and sufficient, though perhaps small, income, and no one to worry or annoy them. For nurses and others who desire a peaceful, gradual, and happy old age, nothing is so desirable as absolute certainty of the means of livelihood and modest comfort when working days are past.

